# Watershed Encoder–Decoder Neural Network for Nuclei Segmentation of Breast Cancer Histology Images

**DOI:** 10.3390/bioengineering13020154

**Published:** 2026-01-28

**Authors:** Vincent Majanga, Ernest Mnkandla, Donatien Koulla Moulla, Sree Thotempudi, Attipoe David Sena

**Affiliations:** Department of Computer Science, University of South Africa, Preller Street, Muckleneuk Ridge, Pretoria 1709, South Africa; majanvi@unisa.ac.za (V.M.); mnkane@unisa.ac.za (E.M.); thotesg@unisa.ac.za (S.T.)

**Keywords:** augmentation, optimization, watershed segmentation, deep learning

## Abstract

Recently, deep learning methods have seen major advancements and are preferred for medical image analysis. Clinically, deep learning techniques for cancer image analysis are among the main applications for early diagnosis, detection, and treatment. Consequently, segmentation of breast histology images is a key step towards diagnosing breast cancer. However, the use of deep learning methods for image analysis is constrained by challenging features in the histology images. These challenges include poor image quality, complex microscopic tissue structures, topological intricacies, and boundary/edge inhomogeneity. Furthermore, this leads to a limited number of images required for analysis. The U-Net model was introduced and gained significant traction for its ability to produce high-accuracy results with very few input images. Many modifications of the U-Net architecture exist. Therefore, this study proposes the watershed encoder–decoder neural network (WEDN) to segment cancerous lesions in supervised breast histology images. Pre-processing of supervised breast histology images via augmentation is introduced to increase the dataset size. The augmented dataset is further enhanced and segmented into the region of interest. Data enhancement methods such as thresholding, opening, dilation, and distance transform are used to highlight foreground and background pixels while removing unwanted parts from the image. Consequently, further segmentation via the connected component analysis method is used to combine image pixel components with similar intensity values and assign them their respective labeled binary masks. The watershed filling method is then applied to these labeled binary mask components to separate and identify the edges/boundaries of the regions of interest (cancerous lesions). This resultant image information is sent to the WEDN model network for feature extraction and learning via training and testing. Residual convolutional block layers of the WEDN model are the learnable layers that extract the region of interest (ROI), which is the cancerous lesion. The method was evaluated on 3000 images–watershed masks, an augmented dataset. The model was trained on 2400 training set images and tested on 600 testing set images. This proposed method produced significant results of 98.53% validation accuracy, 96.98% validation dice coefficient, and 97.84% validation intersection over unit (IoU) metric scores.

## 1. Introduction

Breast cancer (BC) is the most common kind of cancer, especially among women, and if left untreated, it is fatal. Therefore, early diagnosis is key for cancer patients. Recently, cancer detection methods have improved with many utilizing image-based approaches to visualize/screen, and detect cancerous lesions. These include mammography, tomography, breast ultrasound, computed tomography (CT), and magnetic resonance imaging (MRI), among others [1].

Early diagnosis of BC depends largely on the cancerous lesion metastasis to the nuclei and tissue glands. Conventionally, clinicians used the Bloom–Richardson grading system to determine how cancerous lesions morph into nuclei cells, the extent of metastasis, and the increase in the number of these lesion cells [2]. This diagnosis often begins with a deep learning-screened image analysis [3] to detect the presence of cancerous lesions.

However, in some instances, the chosen method produces an indeterminate result. In this case, other methods such as biopsy and hematoxylin and eosin (H&E) analysis of images are used to provide an accurate diagnosis. The H&E staining procedure normalizes and accentuates the nucleus and tissue regions in a histology image. The image contrast is then adjusted to highlight and extract the nucleus part from the background and other non-interesting regions [4]. Traditionally, digital medical image analysis used time-consuming manual methods and inexperienced pathologists. Hence, the introduction of computer-aided (CAD) systems presents more accurate quantitative analysis of histology images.

Recently, machine learning approaches have been utilized for histology image analysis, especially deep learning-based methods, thus increasing the efficacy of histopathology diagnosis [5]. The authors in [6] propose the prediction of the histological grade of prostate cancer via convolutional neural networks (CNN).

Additionally, authors in [7] presented the segmentation of H&E-stained nuclei images via a deep learning-based technique. Its originality is that the segmentation task is similar to regression in distance maps. This fact enables the network to learn and handle intersecting nuclei objects. Consequently, Ref. [8] applied deep learning (DL) methods to tackle digital pathology issues and performed a result analysis of these methods, and compared them with state-of-the-art feature-based methods.

There are several DL networks for breast cancer segmentation and detection tasks. The Pyramidal Feature Aggregation ScanNet (PFA-ScanNet), presented by [9], is a neural network that detects the spread of breast cancer lesions. The authors in [10] present a deep learning framework on the U-net architecture that utilizes MobileNetV2 and VGG12 model encoders for biomedical image semantic segmentation. Their approach integrates the two pre-trained models, having been fine-tuned with the U-net architecture to segment breast ultrasound images. This study provides the following contributions:

- The Albumentations method is an augmentation technique proposed to artificially increase the size of the dataset.
- Morphology operations, erosion, dilation, and distance transformation, are used on the masked augmented images to highlight the ROIs. The morphology operations and data enhancement procedures are handled in the pre-processing phase of this study.
- The connected component analysis method is used on the highlighted ROIs to group components with similar intensities and assign them their respective labeled binary masks.
- The watershed filling method is then applied to the labeled binary masks to separate nuclei objects and their boundaries, thus resolving the issue of edge inhomogeneity, and the issue of intersecting nuclei objects before model processing.
- The WEDN model is optimized by the use of weight regularization techniques such as dropout, early stopping, and learning rate reduction on a plateau to improve the performance of the model. The number of residual convolution blocks is reduced in both the encoder and decoder paths, reducing the number of model parameters used, thus increasing the processing speed. Additionally, the introduction of 2DTranspose provides learnable upsampling in the decoder path. Furthermore, introducing two Conv2D blocks at the bottleneck layer captures and maintains feature spatial resolution in their deep learning.
- WEDN uses skip connections to avoid the loss of important spatial feature information in an image during down-sampling.
- To accurately segment the nuclei, thresholding is applied as a post-processing technique to refine the boundaries of the carious nuclei lesions objects.

## 2. Related Works

Most deep learning-based models for image segmentation tasks use techniques related to the encoder–decoder architecture. Essentially, an encoder–decoder process for image segmentation can be categorized as either a general segmentation or medical/biomedical segmentation technique [11]. The authors in [12] propose a novel segmentation algorithm that learns a deep deconvolution network on top of the convolutional layers borrowed from the VGG16-layer network. The deconvolution network consists of deconvolution and pooling layers, which highlight class labels according to their pixels and predict segmentation tasks.

Another instance of the encoder–decoder approach is the semantic pixel-wise segmentation (SegNet) proposed by [13]. This approach consists of an encoder–decoder network and then a pixel-wise classification layer. The decoder network maps the low-resolution encoder feature maps to generate full input resolution maps for pixel-wise classification. The novelty of SegNet lies in how the decoder upsamples lower resolution feature maps via pooling indices in the max-pooling step of the corresponding encoder to perform non-linear upsampling. This step eliminates the need for learning to perform upsampling.

The paucity of labeled benchmarked training datasets and the need for significant accuracy segmentation increase the need to have a dedicated neural network for biomedical images. Hence, U-Net [14] was introduced, which proposed a U-shaped encoder–decoder architecture that comprises two symmetric paths, namely the contracting and expanding paths. The network can train end-to-end using a small number of images, thus providing significantly higher accuracy than other methods with high-performance segmentation results. There has been an increase in U-Net-related research since its inception, especially for applications targeting medical and biomedical image segmentation tasks. The authors in [15] present some of the U-Net variant models, such as Dense U-Net, U-Net++, ADversarial U-Net, Inception U-Net, and Attention U-Net, among others, that have shown significant improvements on specific medical and biomedical images.

The utilization of the U-Net architecture is significantly appreciated in breast cancer segmentation and detection tasks. Apart from the aforementioned limitation of labeled medical images, DL methods are preferably used in digital pathology for nucleus recognition, segmentation, and classification. However, the nucleus complex features limit their practical use in clinical setups, thus resulting in less accuracy, high computational costs, and a lack of generalizability across various datasets.

Several methods have been proposed to resolve some of these issues. Authors in [16] present the Breast U-shaped Network that consists of spatial and channel attention methods to the segmentation process. Residual blocks and skip connections are included in the architecture to aid extraction of features and in reconstructing the output. We proposed the densely convolutional Breast U-shaped Network (BU-NET) framework to overcome the aforementioned issues. The study employs BU-NET’s spatial and channel attention methods to enhance segmentation processes. Including residual blocks and skip connections in the BU-NEt architecture enhances feature extraction and reconstructs the output, thus detecting and distinguishing nuclei in histology images.

Nuclei cell segmentation is quite a challenging task due to cell morphology, the staining procedure used, and cell topological arrangement in various histology images with different image contrasts. A residual-inception-channel attention-U-Net (RIC-U-Net) proposed by [17] that utilizes the use of residual blocks, multi-scale, and channel attention in its architecture to segment nuclei in histology images. Segmentation of nuclei objects can also be enhanced using U-Net with backbone architectures. The authors of [18] present such a method that enhances the U-Net architecture by using ResNet-34 as an advanced backbone to improve the segmentation of the nucleus. The performance of the proposed model is evaluated and compared with the standard U-Net and its other variants using datasets with augmented nuclei masks.

Segmentation of intersecting nuclei objects due to their morphology in histology images is also a difficult task. A DenseRes-Net model proposed by [19] integrates dense blocks at the end of the encoder block of the U-Net, which focuses on extracted features from previous layers of the model. The model also uses residual connections with atrous blocks instead of skip connections to reduce the semantic gap between the encoder and the decoder blocks. The proposed model also utilizes a distance map and binary threshold methods to highlight nuclei centers and boundary/contour information in the images, respectively.

The manual process of histopathological analysis is quite time-consuming and limited by the quality of images, gland specimens, and pathologists’ experience. The authors of [20] present a review study that trains various deep convolutional neural networks on histology images to segment nuclei of the breast on diverse types of tissues. The performance of these methods was evaluated to determine whether transfer learning aids in segmenting various regions of invasive diseases on the given histology images.

Other challenges in segmenting nuclei images include the wide distribution of cell cluster structures and limited annotated datasets. The study by [21] proposes the utilization of the standard [14] U-Net model in segmenting breast H&E images. The refined segmentation image output is thresholded, and morphology opening, erosion, dilation, and smoothing operations are used to refine the final result.

In recent times, the analysis of histology images and their tissue structures via artificial intelligence has been one of the main methods utilized for cancer diagnosis. Hence, a study by [22] evaluated and compared the performance of various deep learning methods, such as U-Net, ResNet-1001, and Inception-U-Net, among others, on their ability to segment nuclei objects on tissue slides from different organs on several benchmarked datasets. Analysis of image pixel intensity and color distribution was also conducted, and various augmentation methods were applied before training the models. The U-Net model provided significant results after training and testing compared to the other deep learning methods evaluated in the study.

Nuclei segmentation and the counting of nuclei with complex structures play a pivotal role in cancer detection and identification. Authors in [23] present the WaveSeg-U-Net model to segment cancerous intersecting-nuclei objects. The model uses residual blocks for feature extraction in each level of the encoder and decoder. Discrete wavelet transform (DWT) alongside max-pooling is used in the downsampling path, while the inverse DWT is used to regenerate the original images during the upsampling path. Furthermore, atrous spatial channel pyramid pooling (ASCPP) is used to extract high-level features, and watershed transform is used as a post-process technique to identify and accentuate intersecting nuclei objects and count them to assist pathologists.

## 3. Proposed Method

In this section, we meticulously explain the materials and methods used to carry out nuclei segmentation on breast cancer histology images.

### 3.1. Materials

#### Dataset

**Figure 1:**
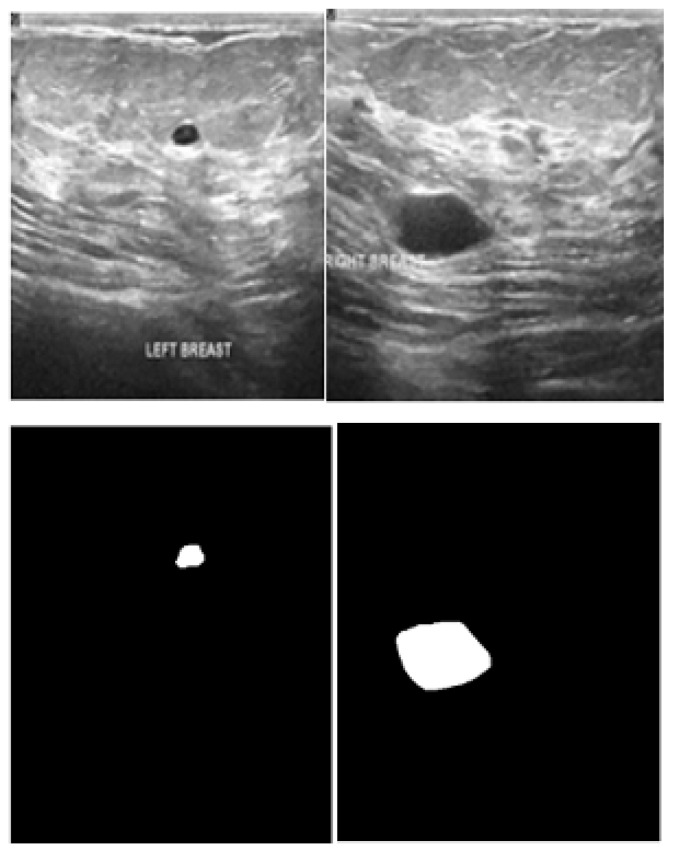
First column: original images. Second column: original masks.

This study uses a 221-image–mask pair dataset of supervised BC histology images that is publicly available Kaggle dataset repository. These image–mask pairs were of different sizes, such as (678 × 571), but all were resized to (128 × 128), and were all in PNG format. Figure 1 shows a sample of the images in the dataset with their corresponding masks.

### 3.2. Method

**Figure 2:**
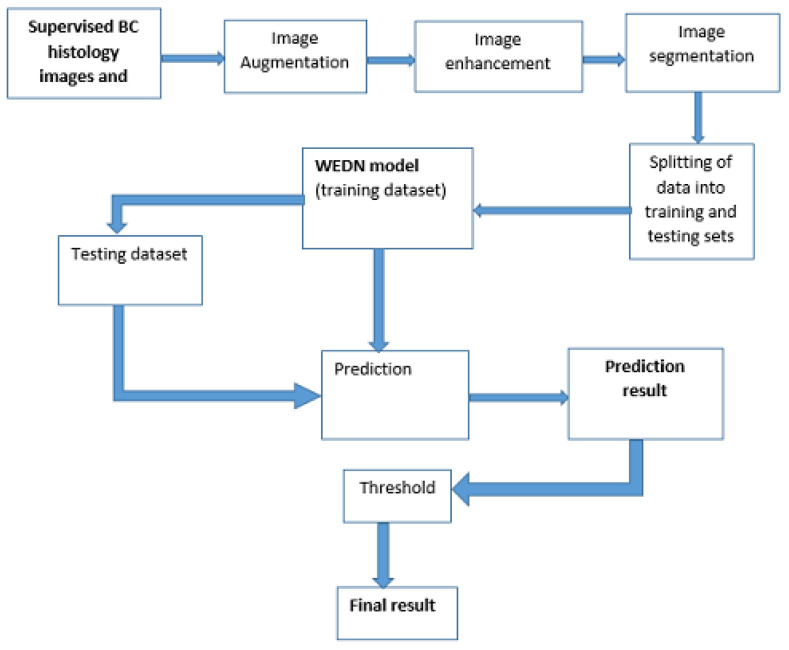
Flowchart of the proposed segmentation method.

Figure 2 shows the steps involved in the proposed method.

This proposed method segments BC histology images by utilizing watershed image segmentation as a pre-processing step to highlight image features, which are then evaluated via the encoder–decoder model network. The proposed hybrid method is summarized by the following steps.

#### 3.2.1. Data Augmentation

Data augmentation is a preferable method for artificially increasing the diversity and quantity of labeled training image sets through using transformations that maintain corresponding output labels. Although most DL methods use image augmentation, they are sometimes limited to basic transformations such as rotation, flipping, scaling, and cropping. Furthermore, model processing speed varies in existing image augmentation libraries. The authors in [24] present Albumentations, a fast and flexible library for image augmentation with several specific image transformation operations.

**Figure 3:**
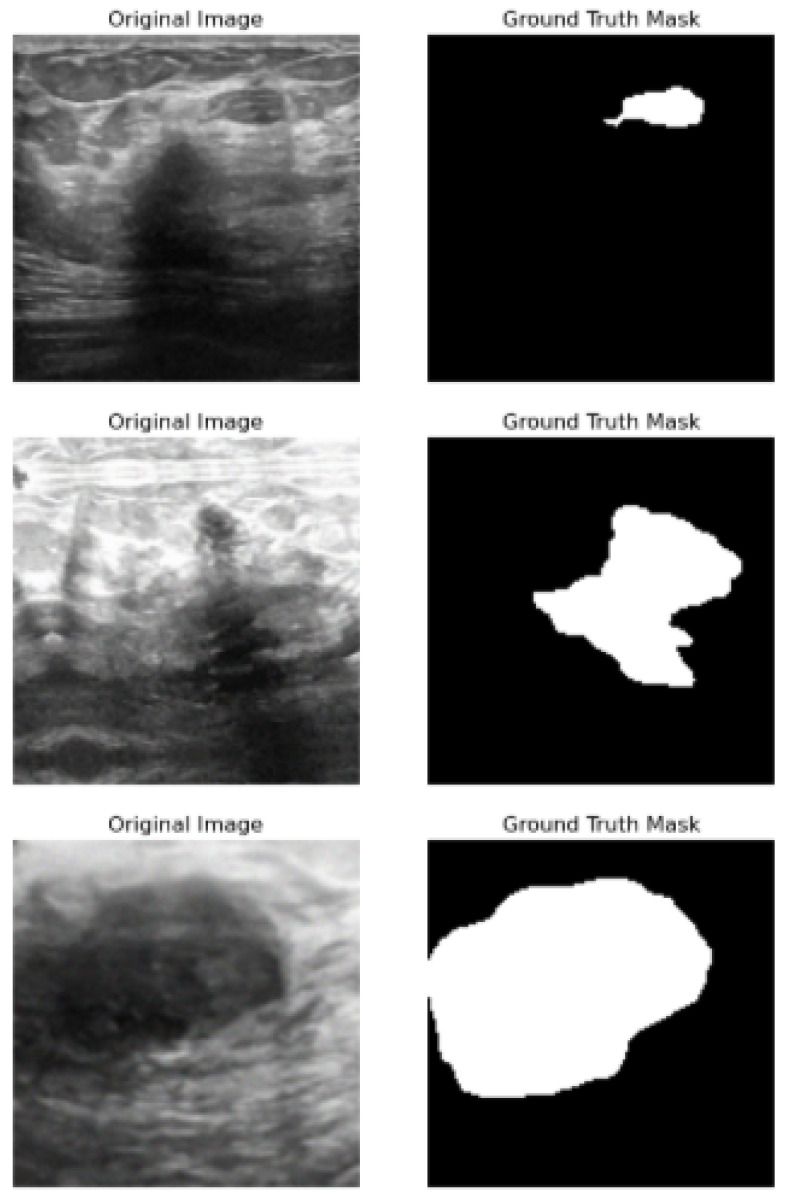
Image–mask pairs after Albumentations.

This study employs Albumentations to augment both our images and mask files. The augmentation transformation used herein is affan ine transform, which consists of Gaussian noise, random brightness contrast, resizing, horizontal flip, hue, saturation value, and composition. With these transformations, the original dataset, comprising 221 images and 221 masks, produces a 3000-image–mask pair dataset. Figure 3 shows the resultant image–mask pair augmented dataset after the application of Albumentations.

#### 3.2.2. Image Enhancement

Consequently, the image–mask pair augmented dataset with 3000 images is enhanced via various data pre-processing and morphology operations to create an auxiliary watershed dataset. The process of image enhancement assists the improvement of image brightness, contrast, and scaling to compensate for the non-uniformity from the data augmentation step. Image enhancement in this study is handled via thresholding, morphology operations, and distance transformation.

##### Thresholding

**Figure 4:**
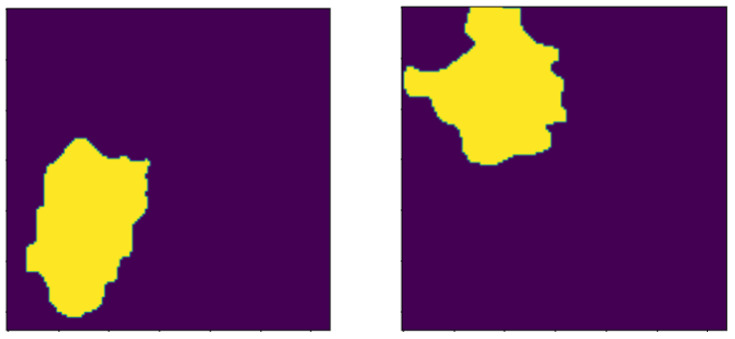
Images after Otsu and binary thresholding.

OTSU thresholding combined with binary thresholding is used to obtain the ROI on the BC histology image. This is shown in Figure 4.

##### Morphology Operations

**Figure 5:**
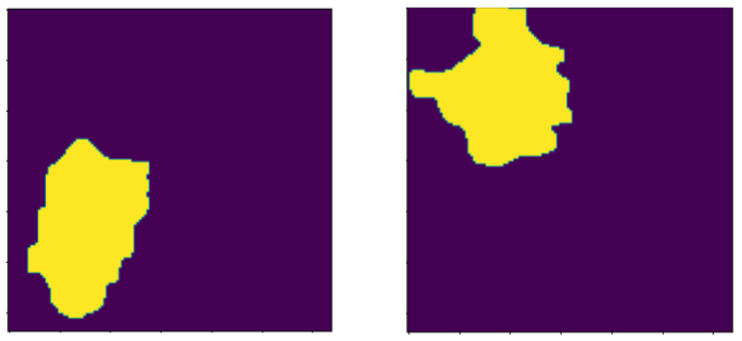
Images after opening morthe phology operation.

**Figure 6:**
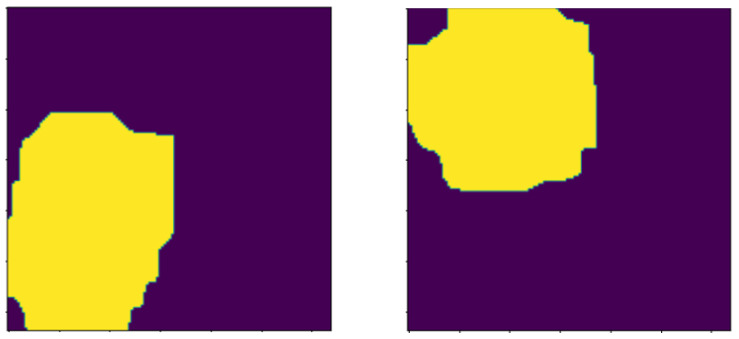
Images highlighting the sure background after the dilation morphology operation.

These operations include dilation morphology to remove noise, overlapping edges, and extract unwanted regions on the histology images. The opening operation is used to remove residual noise to help distinguish the sure background from the foreground in the image. The background and foreground regions are distinguished via diminishing and accentuating image pixels and the edges. Similarly, these operations also highlight the unknown area between the background and the foreground. Figure 5 and Figure 6 visualize image results after the application of the opening, dilation, and distance transformation morphology operations.

##### Distance Transform

**Figure 7:**
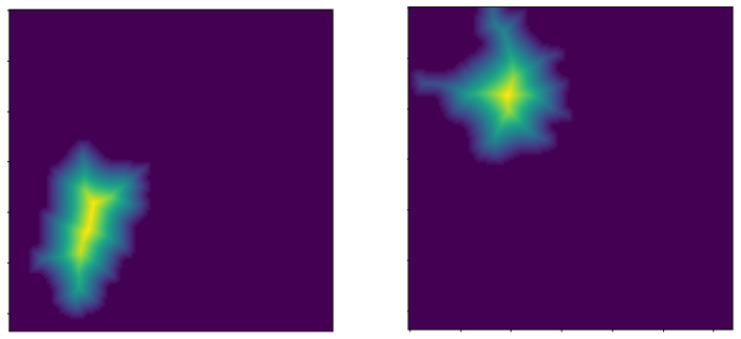
Images highlighting the sure foreground after dithe stance transformation morphology operation.

This method separates nuclei objects from other non-nuclei regions in the image. The resultant region after removing the remaining uninterested ROIs is the sure foreground. This is illustrated in Figure 7.

#### 3.2.3. Segmentation

##### Connected Components

**Figure 8:**
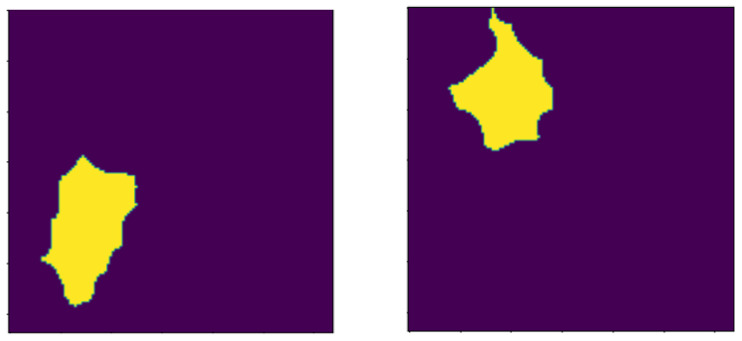
Resultant image ROIs after application of the connected components analysis method.

The obtained image ROIs from the distance transformation operation are then grouped according to those image pixels with similar intensities, with their neighborhood pixels as either darker (background) connected components or brighter (foreground) connected components. These connected components are binary masks that label the sure foreground and sure background regions with corresponding positive numbers, and the unknown region with 0 in an image. The resultant images from the application of connected components are illustrated in Figure 8.

##### Watershed Segmentation

**Figure 9:**
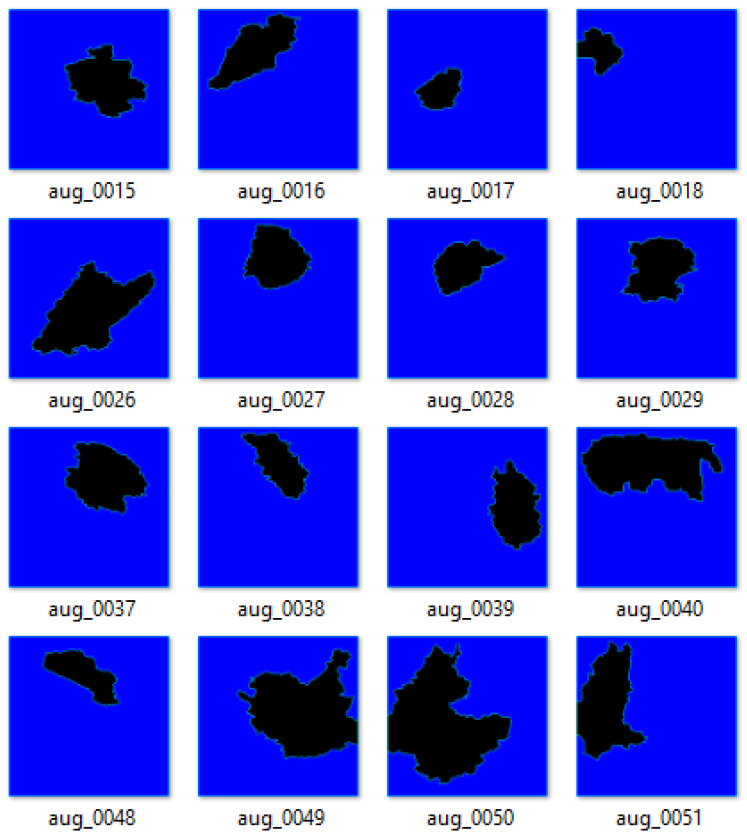
Watershed masked images dataset after application of the watershed segmentation method.

The watershed transform is then subsequently implemented on the obtained ROIs from the connected component method phase. It clearly isolates and maintains the nuclei ROI objects and their edges on the image. The resultant image is color-coded (masked) to highlight image regions such as the background, the foreground (ROI), and the edge boundaries. These watershed masked images form a new folder that has 3000 segmented images. We now have three datasets, namely the original, masked, and watershed masked datasets, with 3000 images each. Figure 9 visualizes the final image results after the application of the watershed segmentation method.

##### Model Architecture

One of the main causes of misclassifications of nuclei objects in histology images is edge/boundary inhomogeneity. The proposed WEDN segmentation model resolves this issue by accurately identifying and highlighting the nuclei boundaries on BC histology images. The proposed WEDN model utilizes the 128 × 128-sized images from both the image and watershed mask augmented datasets as input.

The proposed model employs convolutional residual blocks as feature extractors/learnable layers. It also uses a max pooling 2D layer in the encoder path of the model. Additionally, the bottleneck layer in the proposed model acts as a bridge between the encoder and decoder paths. Further convolution blocks are used in the decoding path of the model to reconstruct images to their original form without losing any information.

**Feature Extraction/Downsampling:** DL network models are used to extract both local and global features. In this study, the downsampling path consists of four convolution blocks, each with two convolution layers (DoubleConv) with the same padding, a rectified linear unit (ReLU) activation function, and a dropout rate value incremented with each subsequent residual block. The kernel filter size at each block is 3 × 3, with a stride of 2 and 2 × 2 maxpooling, whose size becomes the input of the next block for further convolution. The downsampling path aids the learning of image features and hierarchical representation. This actually reduces the input image size while increasing the depth or number of feature maps towards the bottleneck layer.

Equation (1) highlights the additive method (+) of both the skip/stride connection and the convolutional layers of the residual blocks. Here, *w* represents the weight vector, *x* the number of inputs, and *b* the bias associated with the input. The additional *x* represents the additive property of the stride connection and makes the model capable of extracting rich features via feature maps of previous layers.

(1) H(x)=f(wx+b)+x

**Max-Pooling:** In this proposed model, pooling layers are used in the encoder path to reduce image dimensions while extracting global features for the succeeding layers deeper into the model.

(2) $$Z_{i,j}=\max\left\{Y_{m,n}\;|\;m\in \left[2i,2i+1\right],\;n\in \left[2j,2j+1\right]\right\}$$

Given that *Y* is a feature map of shape (H, W) and f is the pooling window (2 × 2), each (2 × 2) block is replaced by the maximum value. In our case, stride = 2; thus, if input = (H, W), then output = (H/2, W/2).

**Bootleneck Layer:** In this study, this layer forms the end of the encoder path and consists of two convolution layers (DoubleConv). The first convolution layer captures extracted features and maintains image spatial resolution, while the next convolution layer enables deeper model learning of features.

(3) $$c_{5}=\sigma \left(W_{2}*\left(D\left(\sigma (W_{1}*X+b_{1})\right)\right)+b_{2}\right)$$

Here, *X* is the input feature map from the previous layer from the encoder path, $W_{1},W_{2}$ are weights for the two convolutional layers in the bottleneck layer, $b_{1},b_{2}$ are bias terms, σ is the ReLU activation function, (D) the dropout layer function, and ∗ is the 2D convolution operator.


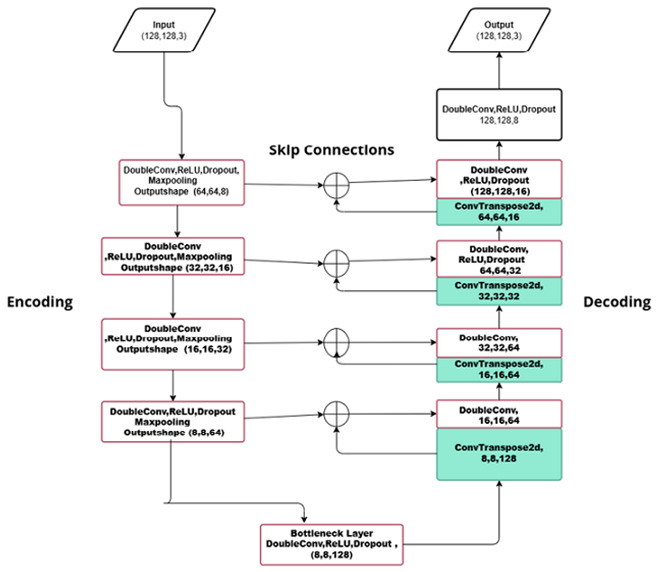


**Learnable Upsampling:** This proposed model uses conv2DTranspose to perform upsampling in the decoder path. It provides trained and learnable upsampling by increasing the image spatial size while reducing its depth, contrary to the encoder path. The resultant feature maps are combined (concatenated) with encoder outputs from corresponding layers via skip/stride connections. Stride connections restore lost spatial information during downsampling. The convolution layers at each decoder step upsample, concatenate, and refine their respective feature information.

(4) $$Z_{i}^{\mathrm{up}}=\sigma \left(W_{i}^{T}*Z_{i-1}+b_{i}^{T}\right)\;(\mathrm{Transpose}\;\mathrm{conv})$$

(5) $$Z_{i}^{\mathrm{cat}}=Z_{i}^{\mathrm{up}}\|S_{i}\;(\mathrm{Concatenate}\;\mathrm{with}\;\mathrm{skip})$$

(6) $$Z_{i}^{\left(1\right)}=\sigma \left(W_{i}^{\left(1\right)}*Z_{i}^{\mathrm{cat}}+b_{i}^{\left(1\right)}\right)\;(\mathrm{First}\;\mathrm{conv})$$

(7) $$Z_{i}^{\left(2\right)}=D_{i}\left(Z_{i}^{\left(1\right)}\right)\;\left(\mathrm{Dropout}\right)$$

(8) $$Z_{i}=\sigma \left(W_{i}^{\left(2\right)}*Z_{i}^{\left(2\right)}+b_{i}^{\left(2\right)}\right)\;(\mathrm{Second}\;\mathrm{conv})$$

(9) $$\hat{Y}=\mathrm{softmax}\left(W^{\mathrm{out}}*Z_{n}+b^{\mathrm{out}}\right)\;(\mathrm{Final}\;1\;\times \;1\;\mathrm{conv}\;\mathrm{for}\;\mathrm{segmentation})$$

Here, each decoder block is listed as i∈1,2,...,n, $Z_{n}$ and is the output of the final decoder layer, $W^{out}$ are the weights of the output convolution layer, $b^{out}$ are the biases of the output 1 × 1 convolution layer, and $\hat{Y}$ is the final predicted segmentation map.

The proposed model (WEDN) learns to identify nuclei ROI objects based on learned features from trained data. It learns by capturing both discriminative and spatial features of the provided labeled training data (image–watershed masks). Deeper model processing splits the image classes into 0 for background, 1 for nuclei objects, and 2 for edges/boundaries. The model learns to map the input image to the segmentation map by optimizing a loss function that compares the predicted segmentation output with the ground truth(mask images).

##### Thresholding

The thresholding technique is used as a post-processing approach to convert the resultant output image (predicted mask) into a binary image via a threshold value. This compares each pixel value in the resultant output image with the threshold value. This study uses the threshold value to isolate the nuclei, backgrounds, and edges based on differences in intensity values. After several iterative experiments with different threshold values(0.5, 0.2, 0.4, 0.3), this study settled on the best threshold value of T = 0.35.

## 4. Results and Discussion

This study utilized the WEDN and an optimized U-Net model architecture to perform nuclei segmentation on augmented supervised breast cancer histology images. The experiments were carried out on 3000 image–watershed mask pairs, split into 2400 training and 600 testing set images. After several experiments, we evaluated the performance of the WEDN model on our validation set, and the results were not convincing. Therefore, we set out to introduce hyperparameter tuning to improve the model performance. Several tuning parameters were utilized, such as learning rate, dropout rate, batch size, and number of iterations/epochs, to capture the nuclei segmentation of histopathological BC images. WEDN is a lightweight model with 485,826 parameters.

Early stopping and a reduced learning rate were introduced after it was realized that the model had reached a plateau with a constant learning rate. Hence, these tuning parameters were integrated into the model to monitor validation loss. Additionally, after more experiments, we also witnessed that as the number of epochs increased, from 20 to 35, 50, and 75, it resulted in even better evaluation metric results. Therefore, in this proposed study, significant results were optimally obtained at 100 epochs. Consequently, the model also settled on the Adam optimizer with a learning rate of 0.0004, dropout rate (0.1–0.4) on the convolution blocks, the dice + categorical cross entropy loss function, batch normalization, and weight decay. Additional model metrics, such as dice coefficient, IoU score, and accuracy, were also used to measure model performance.

### Evaluation Metrics

The performance evaluation of the proposed model was carried out using the Dice coefficient, intersection over union (IoU), and accuracy evaluation metrics. Equation (10), Equation (11), and Equation (12) describe the IoU, the Dice coefficient, and accuracy evaluation metrics as follows:

(10) $${\mathrm{IoU}}_{k}=\frac{\sum_{i}⊮\left[{\hat{Y}}_{i}=k\;\wedge \;Y_{i}=k\right]}{\sum_{i}⊮\left[{\hat{Y}}_{i}=k\;\vee \;Y_{i}=k\right]},\;k=1,2,\ldots ,K$$

Given that ${\mathrm{IoU}}_{k}$ is for class *k*, ${\hat{Y}}_{i}$ is the predicted class label at pixel *i*, $Y_{i}$ the ground truth class label at pixel *i*, 1[·] is the indicator function (equals 1 if the condition is true), and ∧ is the logical AND (intersection), with ∨ being the logical OR (union) and *K* the total number of classes.

(11) $$\mathrm{Dice}=\frac{2\cdot |\hat{Y}\cap Y|}{|\hat{Y}\left|+|Y\right|}=\frac{2\sum_{i}{\hat{Y}}_{i}\cdot Y_{i}}{\sum_{i}{\hat{Y}}_{i}+\sum_{i}Y_{i}}$$

Here, $\hat{Y}$ is the predicted segmentation mask, *Y* is the ground truth mask, ${\hat{Y}}_{i},Y_{i}\in 0,1,2$. Numerator: 2 × intersection. Denominator: sum of sizes (predicted + true).

(12) $$\mathrm{BC}\;\mathrm{dataset}\;\mathrm{accuracy}=\frac{\sum_{n=1}^{N}\sum_{i=1}^{H}\sum_{j=1}^{W}1\;\left(y_{ij}^{n}={\hat{y}}_{ij}^{n}\right)}{N\times H\times W}$$

**Figure 10:**
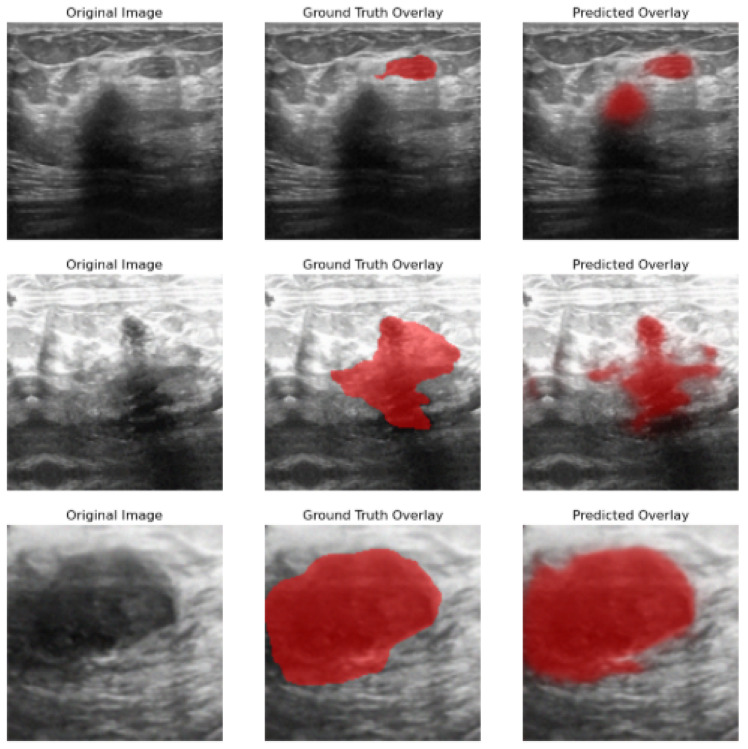
Image output results before weight regularization techniques were applied to the proposed model.

**Figure 11:**
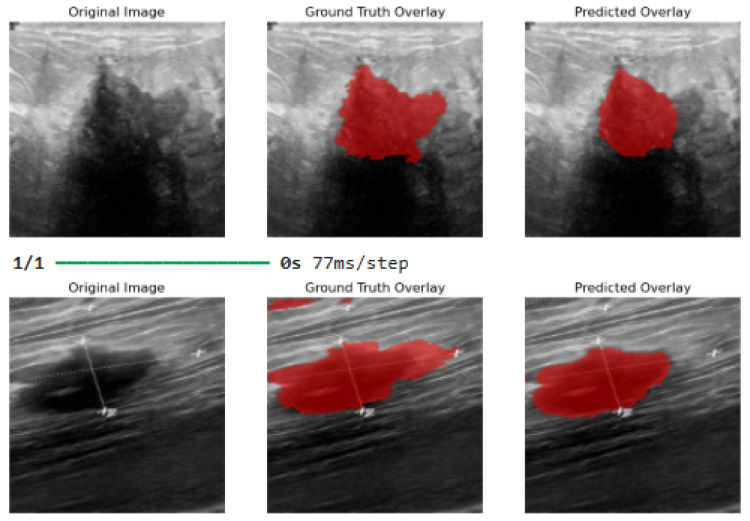
Image output results after 20 epochs and applying model hyperparameter tuning.

Given that, *N* is the total number of images in the dataset, H×W are the image dimensions per pixel, $y_{ij}^{n}$ and ${\hat{y}}_{ij}^{n}$ are the ground truth and predicted label of pixel i,j in image *n*, respectively, and 1(·) is the indicator function. Figure 10 illustrates the original, ground truth overlay, and predicted output image overlay results after application of the proposed method. Figure 11 shows a visual representation of the original, ground truth overlay, and predicted overlay output by the proposed hyperparameter-tuned model after 20 epochs.

**Figure 12:**
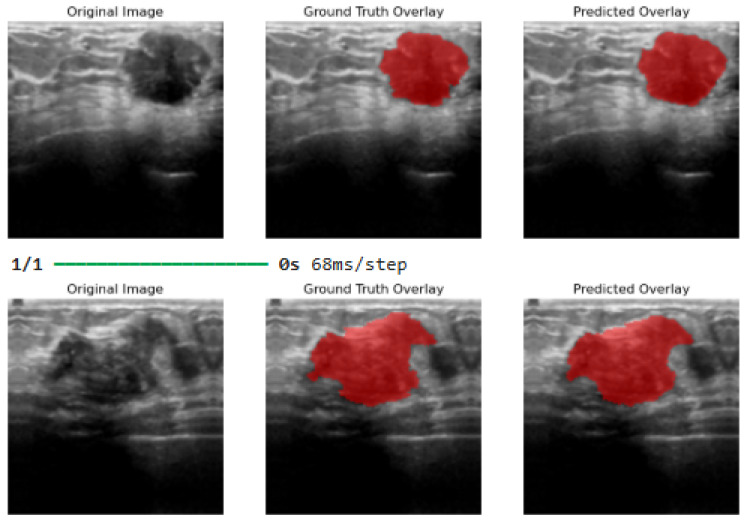
Final image output results after 100 epochs and applying model hyperparameter tuning. Best threshold at 0.35.

Figure 12 shows the final visual results from the hyperparameter-tuned model after 100 epochs. Other weight regularization techniques used for hyperparameter tuning include L2 regularization (weight decay), which reduces the weights of the parameters, thus improving generalization. Dropout is used to drop some activations during training, thus reducing dependency on specific neurons. Additionally, batch normalization was used to stabilize training without compromising memory limits by allowing higher learning rates, thus improving convergence stability (avoiding plateaus). These weight regularization techniques, used collectively, improved the robustness of the proposed segmentation model by reducing overfitting.

Apart from weight regularization techniques, the improvement of results was also attributed to dataset augmentation (albumentations), which increased the dataset size. Morphology operations were also used to highlight BC nuclei ROIs. Image enhancement methods combined with segmentation methods, such as connected component analysis and watershed, were used to resolve intersecting nuclei objects and edge inhomogeneity, respectively. Table 1 presents a comparison between the proposed approach and other state-of-the-art U-Net-related breast histology image segmentation techniques.

**Figure 13:**
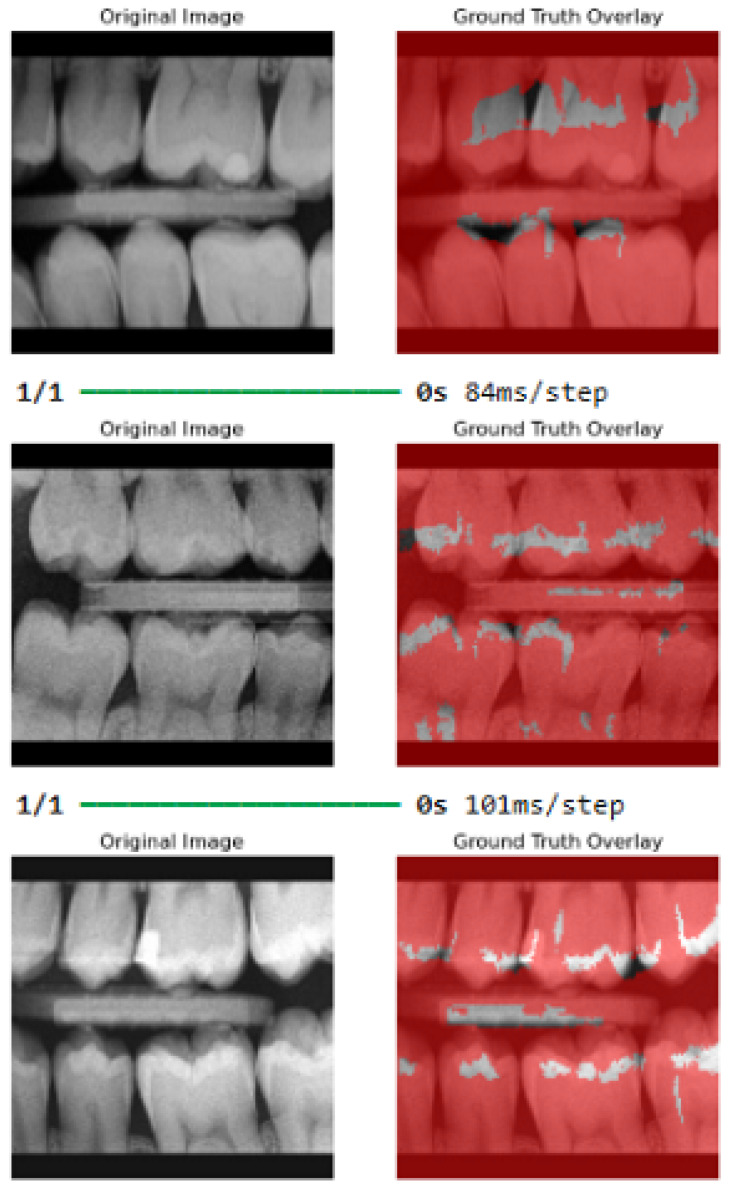
Dental image results after application of our proposed segmentation method.

**Figure 14:**
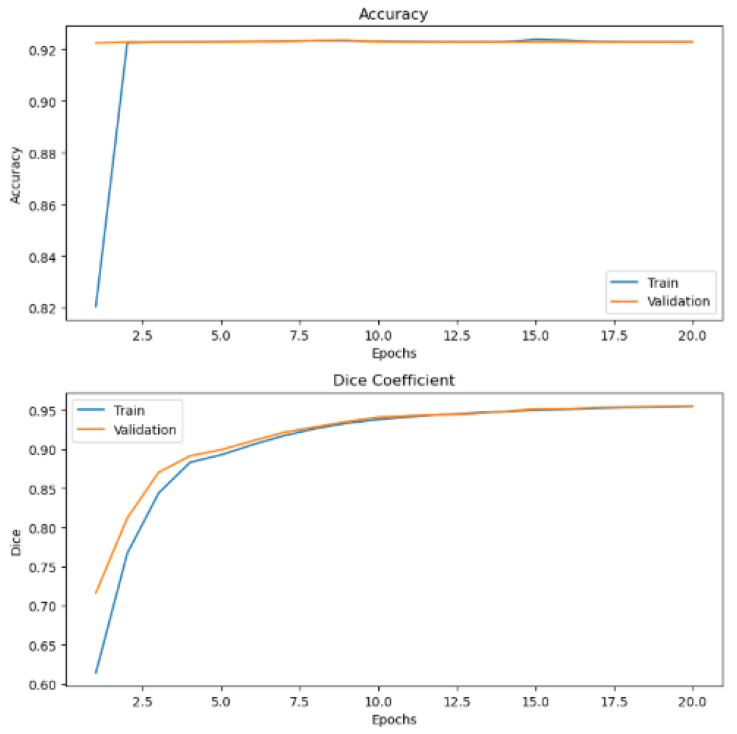
Dental images graph results after application of our proposed segmentation method.

The practicability of our approach has been tested on publicly available dental caries in bitewing radiographs from the Mendeley data dataset repository. This dataset consists of 100 bitewing radiographs that we pre-processed and fed into our WEDN model for processing and further evaluation. Since the dataset did not have masks, we used the watershed segmentation method to create a dental watershed mask dataset with 100 images. The 100-image–watershed mask pair dataset was then augmented to create two datasets of 6000 images each. These augmented datasets were then fed to our WEDN model for training and testing. After model processing, its performance was evaluated, and Figure 13 shows the original images and the ground truth overlay image results. Figure 14 illustrates the training and validation graph curves of the dental caries Mendeley data dataset after applying the proposed segmentation method. This yielded the significant result of a validation dice coefficient of 95.52%, a validation accuracy of 92.28%, and a 91.48% IoU score.

**Figure 15:**
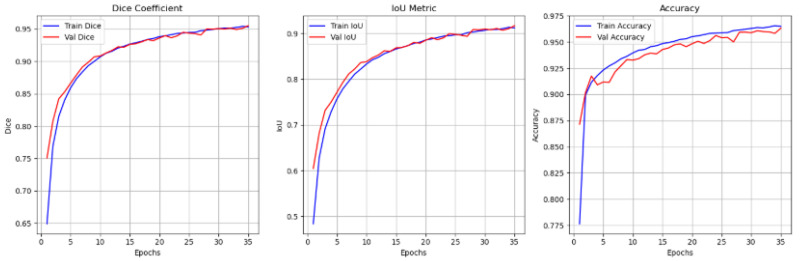
Performance evaluation metrics graph results before weight regularization techniques were applied to the proposed model.

**Figure 16:**
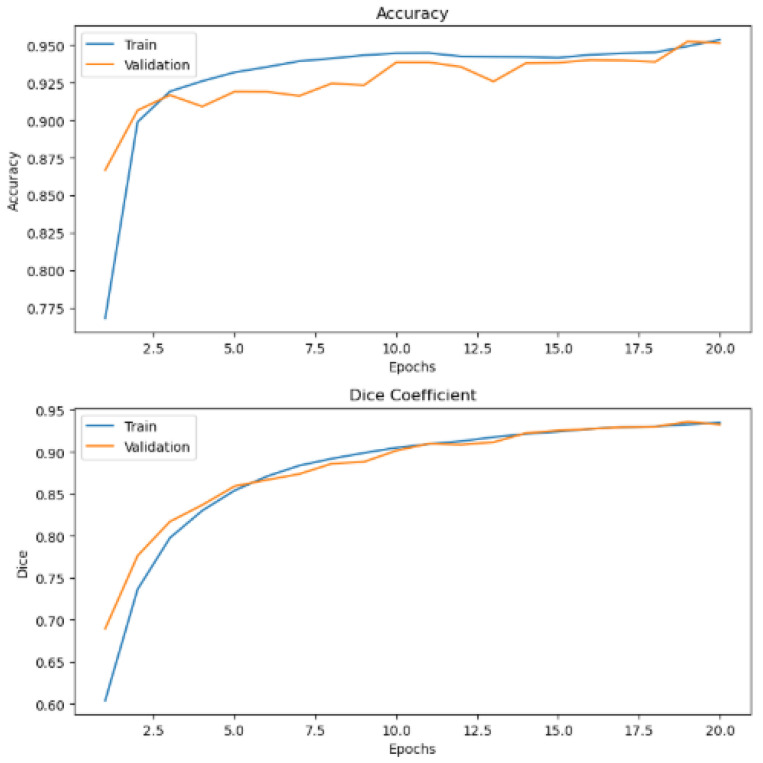
Performance evaluation metrics graph results after 20 epochs and applying model hyperparameter tuning.

**Figure 17:**
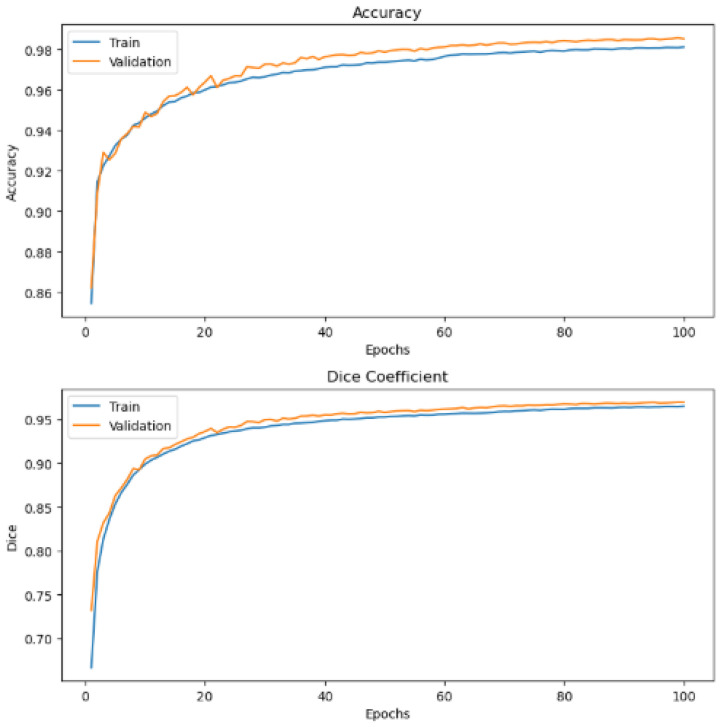
Performance evaluation metrics graph results after 100 epochs and applying model hyperparameter tuning. Best threshold at 0.35.

Figure 15 provides a graphical representation of the various evaluation metrics used: dice coefficient and accuracy of the proposed method before regularization is applied on BC images. Figure 16 and Figure 17 show graph representations of the proposed method after weight regularization techniques are applied. From the graphs, it is quite clear that model overfitting has been resolved.

## 5. Conclusions

This study proposes the watershed encoder–decoder neural network (WEDN) to segment cancerous lesions in supervised breast histology images. The lightweight model architecture comprises four encoder and decoder stages with residual convolution blocks. The model achieved significant results of 98.53%, 96.98%, and 97.84% for validation accuracy, validation dice coefficient, and validation IoU, respectively, outperforming state-of-the-art models. The three metrics were used collectively to ensure meticulous assessment of the proposed model by capturing both local and global features, and to resolve the nuclei objects’ intersection while maintaining and highlighting their boundaries/edges.

We acknowledge differences in the reported metrics of other related studies compared to ours, which may limit direct comparison with the proposed method. Additionally, while this study reports performance metrics such as IoU, dice coefficient, and accuracy, we also acknowledge that it does not include statistical validation measures such as variance and confidence intervals. Future work will incorporate statistical validation to strengthen the robustness and interpretability of our research findings.

Consequently, the proposed model is designed and employed to overcome the limitations of small datasets, segmentation of intersecting objects, and edge inhomogeneity. Notwithstanding the significant results, the study acknowledges that the small dataset of 221 images was expanded to 3000 via augmentation, raising the potential for model overfitting, thus restricting model generalizability. Furthermore, over-segmentation in certain ROI and color inconsistencies arose from image variations from the augmentation step. These issues underscore the importance of utilizing color normalization methods to ensure consistent feature extraction and improve segmentation reliability.

The use of Albumentations as an augmentation improvement technique also resolves dataset size limitations. Pre-processing and image enhancement techniques offer a ripple effect on the extraction and learning of features by the proposed model. For future work, the WEDN model can be improved by incorporating other weight regularization techniques to optimize and improve its robust performance. Additionally, it is important to validate the model on large and more diverse datasets, such as other life-threatening conditions that require medical imaging analysis to evaluate real-world applicability. Consequently, exploring unsupervised learning structures could reduce the model’s dependency on labeled data and may improve its diagnostic depth. These future directions aim to expand the proposed models’ applicability beyond supervised BC segmentation towards broader medical imaging tasks and domains.

## Figures and Tables

**Table 1:** The proposed method’s performance evaluation metrics in comparison with other state-of-the-art methods.

Reference Authors	Methods	Evaluation Metrics	Scores
Dinh et al. [3]	Efficient U-Net++	Recall, F1 score	93.43%
Bhattacharjee et al. [6]	Multi-channel CNN	F1 accuracy	95.1%
Zhao et al. [9]	PFA-ScanNet (Pyramid Feature Aggregation)	Kappa score	90.5%
Rehman et al. [16]	BU-Net (Densely Conv. Breast U-Net)	F1 score	91.8%
Zeng et al. [17]	RIC-U-Net (Residual Inception Channel Attention)	Dice, F1 score	80.08%, 82.78%
CV et al. [18]	U-Net + ResNet-34	IoU, Accuracy	79.5%, 98.3%
Kiran et al. [19]	DenseRes-U-Net	F1 score	90.03%
Mardhatillah et al. [21]	U-Net	Accuracy, IoU	92.70%, 74.54%
Khan et al. [23]	WaveSeg-U-Net	F1 score, Jaccard index	94.0%, 80.0%
**Proposed Method**	**WEDN (Watershed Encoder–Decoder Net)**	**Accuracy, Dice coefficient, IoU score,**	**98.53%, 96.98%, 97.84%**

## Data Availability

The data and code used to support the findings of this study can be obtained from the corresponding authors upon request. The data are not publicly available due to [ethical concerns and limited publicly available datasets].
